# A young adult presenting with granulomatous hepatitis and nephrotic syndrome: A case report

**DOI:** 10.1186/1755-7682-5-29

**Published:** 2012-10-25

**Authors:** Kumarangie Vithanage, Kanpathipillai Thirumavalavan

**Affiliations:** 1 Department of Medicine (Ward 13), Colombo North Teaching Hospital, Colombo, Sri Lanka

## Abstract

Amyloidosis is a rare disease characterised by the deposition of insoluble extracellular fibrillar proteins in various tissues of the body. The pattern of manifestation is organ dependent and also on whether the disease is localised or systemic, primary or secondary. Primary systemic amyloidosis is a disease of adulthood. In reported cases, the mean patient age of onset is 65 years. We report a case of a young adult who presented with jaundice and leg oedema which ultimately found to have granulomatous hepatitis and nephrotic syndrome secondary to systemic amyloidosis. The purpose of this case report is to reiterate the importance of a high index of suspicion in considering amyloidosis in such presentations regardless of the presenting age.

## Introduction

Amyloidosis is a rare disease characterised by the deposition of an insoluble extracellular fibrillar protein in various tissues of the body [1].

It is classified according to the type of amyloid protein deposited. The four categories are primary (immunoglobulin light chain [AL]), secondary (serum protein A, produced in inflammatory conditions [AA]), hereditary and renal failure type. Amyloidosis is further classified as localized (amyloid deposits only in a single tissue type or organ) or, most common, systemic (widespread amyloid deposition) [2].

Primary systemic amyloidosis involves the deposition of insoluble monoclonal immunoglobulin light (L) chains or L-chain fragments in various tissues, made by plasma cells in the bone marrow. These L-chains are secreted into the serum. It's a disease of adulthood with the mean patient age of onset being 65 years in reported cases [3].

Renal disease is the commonest manifestation and presents as progressive renal impairment with proteinuria [4].

The input of amyloid protein into tissues far exceeds its output, so amyloid build up inexorably proceeds to organ dysfunction and ultimately organ failure and premature death [2].

## Case presentation

A 38 year old male presented with bilateral leg swelling and frothy urine along with yellowish discolouration of eyes for 1 week duration. Furthermore he had been anorexic and there was progressive weight loss during the last 3 months. He did not have fever. Urine output had been normal and there was no hematuria. There were no features of SLE or vasculitis and patient denied long term use of nephrotoxic or hepatotoxic drugs. He has not had hepatitis in the past and neither admitted any high risk sexual behaviours. There was no family history suggestive of liver or renal disease or amyloidosis. He was a lifelong non smoker and had never consumed alcohol.

Physical examination revealed an ill looking afebrile patient with deep icterus and bilateral pitting ankle oedema extending proximally up to the level of mid leg. He had no peripheral stigmata of chronic liver disease or evidence of encephalopathy. There was no evidence of lymphadenopathy, kayser fleischer rings or vasculitis. There was tender firm hepatomegaly without splenomegaly along with moderate ascites. Cardiovascular, respiratory and neurological examination was normal.

The urine full report showed albuminuria and bile in the urine. Full blood count remained normal throughout. The blood picture showed round macrocytes with few target cells without rouleaux formation. The initial ESR was 45 mm/h and the CRP was 5.8 mg/l.

The liver biochemistry showed progressive worsening of hepatocyte function with predominant rise in GGT and Alkaline phosphatase (Table 1). The INR was 1.2 initially but increased up to 2.2 subsequently.

**Table 1:** Liver and renal biochemistry time-line

**Test**	**Day 1**	**Day 5**	**Day 10**
AST (U/L)	70	89	103
ALT(U/L)	86	87	82
SBR (mg/dl)	1.8	2.2	3.5
GGT (U/L)	900	1024	1218
ALP (U/L)	3900	4180	4196
S Protein (g/dl)	5.2	4.2	3.9
ALB (g/dl)	2.8	2.3	2.0
GLB (g/dl)	2.1	1.9	1.9
S Cr	0.8	0.93	1.8

The serum electrolytes and renal function were within normal limits initially but was deteriorating during the hospital stay. Urinary protein: creatinine ration was 4.2. Urine for Bence Johns Protein was negative and the serum and urinary protein electrophoresis were normal without any abnormal monoclonal band. Skeletal survey was not suggestive of myeloma.

Serum ferritin and LDH were with normal range and ANA, Rheumatoid factor and Anti-mitochondrial antibody were not detected. Hepatits B and C serology was negative.

The ultrasound scan of the abdomen showed an enlarged liver at 17.5 cm with a coarse echo pattern there was moderate ascites. The intra and extra-hepatic bile ducts were not dilated. There was no splenomegaly. There was evidence of acute renal parenchymal changes and hepatic and renal arterial flow was seen normal upon duplex imaging.

2D Echocardiogram demonstrated restrictive cardiomyopathy with good left ventricular function.

**Figure 1:**
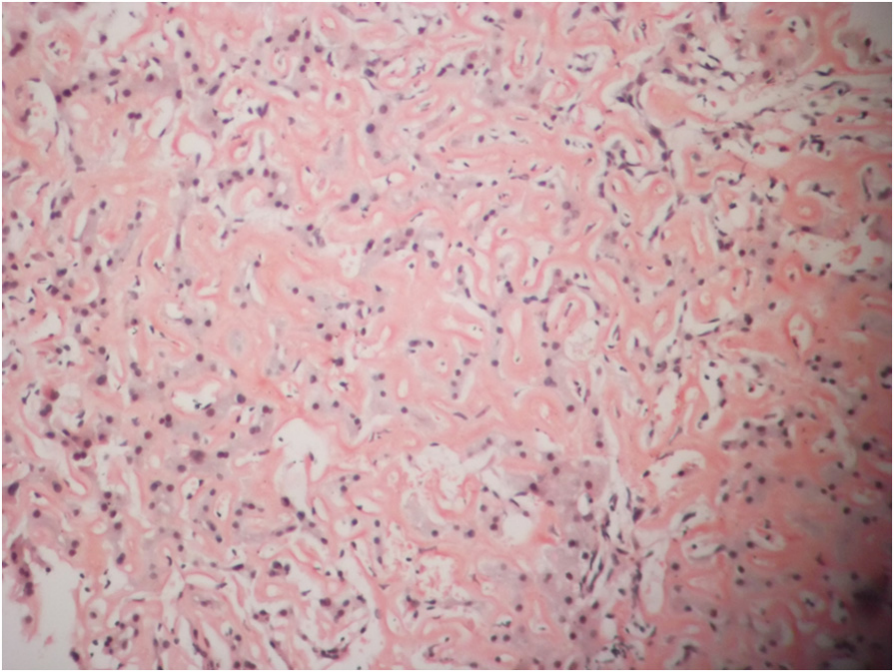
Amyloid deposition of liver as stained with Congo red along with compression atrophy of the liver parenchyma.

**Figure 2:**
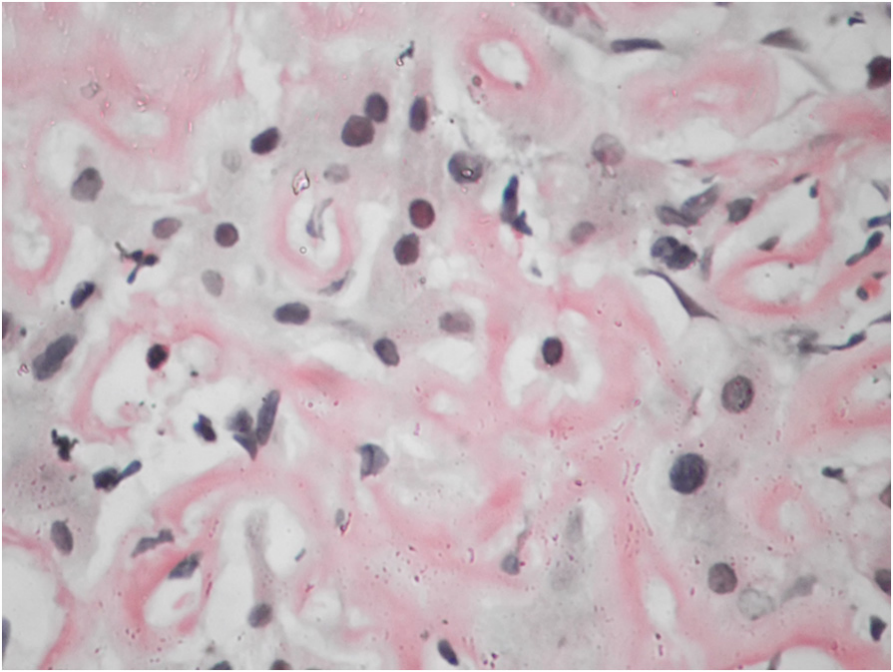
Peritubular and endothelial amypoid deposits as stained with Congo red confirming renal amyloidosis.

Subsequently he underwent liver and renal biopsy. (Figure 1 and 2 respectively).

Biopsies of both the liver and kidney was performed to exclude other differential diagnosis which could co exist as viral hepatitis and lymphoma which may also present as granulomatous hepatitis and nephrotic syndrome.

Patient did not consent for bone marrow biopsy. He was treated with liver failure regimen with salt restriction. Diuresis with furosemide and spironolactone was commenced. With the establishment of the diagnosis of amyloidosis it was decided to start him on high dose prednisolone 50 mg daily with melphalan 50 mg daily.

However patient rapidly deteriorated and succumbed after 2 days of commencing above medication due to acute liver failure and hepato-renal shut down.

## Discussion

Amyloidosis is not a single disease but a term for diseases that share a common feature: the extracellular deposition of pathologic insoluble fibrillar proteins in organs and tissues. In the mid-19th century, Virchow adopted the botanical term “amyloid,” meaning starch or cellulose, to describe abnormal extracellular material seen in the liver at autopsy. Subsequently, amyloid was found to stain with Congo red, appearing red microscopically in normal light but apple green when viewed in polarized light. Almost a century after Virchow's observations, the fibrillar nature of amyloid was described with the use of electron microscopy and the characteristic beta-pleated–sheet configuration, now believed to be responsible for the typical staining properties, was identified [5].

Primary systemic or light chain amyloidosis (AL) is characterized by a clonal population of plasma cells in the bone marrow that produce monoclonal light chain of kappa or lambda type [6]. Amyloid fibrils deposit in organs, progressively interfering with organ structure and function. Commonly affected organs include the heart, kidneys, gastrointestinal tract/liver or the peripheral or autonomic nervous system.

In Sri Lanka apart from Congo red staining there are no facilities available for amyloid light chain detection. In this case there was evidence of multi system involvement mainly the liver, kidney and the heart without any detectable evidence of multiple myeloma although the bone marrow biopsy could not be performed. Furthermore he did not have any condition that could give rise to secondary amyloidosis and neither had he had a family history of the same. Thus it's very likely that the patient had primary systemic amyloidosis although light chain detection was not done.

## Conclusion

Amyloidosis, though a rare disease is not uncommon. The need for a detailed and comprehensive patient evaluation cannot be overemphasized. So the pivotal role of a doctor would be to have high index of suspicion in evaluating such patients with multi system disease manifestations and specifically request for Congo red staining of tissue biopsy as it's the only investigatory tool available in our resource poor set up which could identify amyloid deposits.

## Consent

Written informed consent was obtained from the next of kin of the patient for publication of this case report and accompanying images as the patient succumbed prior to submission of the report. A copy of the written consent is available for review by the Editor-in-Chief of the International Archives of Medicine.

## Abbreviations

SLE: Systemic Lupus Erythematosus; CRP: C reactive protein; ESR: Erythrocyte sedimentation rate; AST: Aspartate amino transferase; ALT: Alanine amino transferase; SBR: Serum bilirubin; GGT: Gamma glutamyl transferase; ALP: Alkaline phosphatase; ALB: Albumin; GLB: Globulin; S Cr: Serum creatinine; ANA: Anti nuclear antibody; LDH: Lactate hdehydrogenase.

## Competing interests

The authors declare that they have no competing interests.

## Authors' contributions

KV carried out the literature search and drafted the manuscript; KT did the critical revision for important intellectual content in the manuscript and given the final approval of the version to be published; both the authors read and approved the final manuscript.
